# Modified VEGF-A mRNA induces sustained multifaceted microvascular response and accelerates diabetic wound healing

**DOI:** 10.1038/s41598-018-35570-6

**Published:** 2018-11-30

**Authors:** Naidi Sun, Bo Ning, Kenny M. Hansson, Anthony C. Bruce, Scott A. Seaman, Chenchu Zhang, Michaela Rikard, Christopher A. DeRosa, Cassandra L. Fraser, Maria Wågberg, Regina Fritsche-Danielson, Johannes Wikström, Kenneth R. Chien, Anna Lundahl, Mikko Hölttä, Leif G. Carlsson, Shayn M. Peirce, Song Hu

**Affiliations:** 1 Department of Biomedical Engineering, University of Virginia, Charlottesville, VA 22908 United States; 2 Bioscience Heart Failure Cardiovascular, Renal and Metabolism, IMED Biotech Unit, AstraZeneca, Gothenburg, Sweden; 3 Department of Chemistry, University of Virginia, Charlottesville, Virginia 22904 United States; 4 Cardiovascular, Renal and Metabolism, IMED Biotech Unit, AstraZeneca, Gothenburg, Sweden; 5 Integrated Cardiometabolic Center, Karolinska Institute, SE-141 52 Huddinge, Sweden; 6 Department of Cell and Molecular Biology and Medicine, Karolinska Institute, SE-171 77 Stockholm, Sweden; 7 Department of Drug Metabolism and Pharmacokinetics Cardiovascular, Renal and Metabolism, IMED Biotech Unit, AstraZeneca, Gothenburg, Sweden; 8 Safety & ADME Translational Sciences, Drug Safety and Metabolism, IMED Biotech Unit, AstraZeneca, Gothenburg, Sweden

## Abstract

Capable of mediating efficient transfection and protein production without eliciting innate immune responses, chemically modified mRNA holds great potential to produce paracrine factors at a physiologically beneficial level, in a spatiotemporally controlled manner, and with low toxicity. Although highly promising in cardiovascular medicine and wound healing, effects of this emerging therapeutic on the microvasculature and its bioactivity in disease settings remain poorly understood. Here, we longitudinally and comprehensively characterize microvascular responses to AZD8601, a modified mRNA encoding vascular endothelial growth factor A (VEGF-A), *in vivo*. Using multi-parametric photoacoustic microscopy, we show that intradermal injection of AZD8601 formulated in a biocompatible vehicle results in pronounced, sustained and dose-dependent vasodilation, blood flow upregulation, and neovessel formation, in striking contrast to those induced by recombinant human VEGF-A protein, a non-translatable variant of AZD8601, and citrate/saline vehicle. Moreover, we evaluate the bioactivity of AZD8601 in a mouse model of diabetic wound healing *in vivo*. Using a boron nanoparticle-based tissue oxygen sensor, we show that sequential dosing of AZD8601 improves vascularization and tissue oxygenation of the wound bed, leading to accelerated re-epithelialization during the early phase of diabetic wound healing.

## Introduction

Tissue ischemia due to the lack of proper vascularization is a major complication that underlies a wide variety of devastating diseases, including coronary artery disease, heart failure, critical limb ischemia, and chronic diabetic ulcers with impaired wound healing ability^1–4^. These areas with large unmet medical needs would greatly benefit from novel and effective pharmacological treatments. Targeting the stimulation of angiogenesis, arteriogenesis, and lymphangiogenesis, therapeutic vascular growth has been put forward as a promising strategy for management of such indications^5,6^. Administered in the form of recombinant protein or via naked or adenoviral vector-mediated gene transfer, the vascular endothelial growth factor A (VEGF-A; especially its primary isoform with 165 amino acids, VEGF-A_165_) has shown potential beneficial effects in cardiovascular diseases and wound healing and has received increasing attention^6,7^. However, randomized controlled trials have not lived up to expectations and the clinical efficacy remains inconclusive^8,9^. Although the overall reason(s) for the underwhelming clinical benefit is unclear, possible limiting factors of current VEGF-A therapies include suboptimal pharmacokinetics and insufficient local concentration of the protein, poor gene transfer efficiency, inappropriate timing of dosing, regression of immature vessels, and growth factor-related adverse effects, such as edema and systemic hypotension^5^.

To circumvent these issues associated with recombinant protein and gene transfer-based therapies, chemically modified mRNA has been developed as a novel non-immunogenic and non-integrating modality for efficient and transient expression of target proteins in selected tissues *in vivo*^10,11^. Encoding VEGF-A_165_, AZD8601 is a new, purified and clinical-grade modified mRNA optimized to ensure efficient transfection and protein production with minimal innate immune response^12,13^. Recently, it was demonstrated in the subacute setting of porcine myocardial infarction that intracardiac injection of AZD8601 formulated in a biocompatible citrate/saline vehicle resulted in improved left ventricular function, increased neovessel formation, and attenuated cardiac fibrosis 2 months after injection^12,13^. Moreover, AZD8601 has been brought into clinical testing in patients with type-2 diabetes mellitus and in patients undergoing open heart surgery, with the primary objective to assess the safety and tolerability of this new therapeutic (ClinicalTrials.gov Identifiers: NCT02935712 and NCT03370887). Although promising, the functional pharmacodynamic effects and the potential therapeutic benefits of this emerging therapeutic on the microvasculature needs further characterization *in vivo*.

Here, we quantitatively characterize the dynamic changes in the microvascular structure and function following single or repeated intradermal injections of AZD8601 in the live mouse ear. With the aid of label-free, noninvasive multi-parametric photoacoustic microscopy (PAM)^14,15^, we demonstrate the pronounced, sustained and dose-dependent effects of AZD8601 on the microvasculature through side-by-side comparison against recombinant human VEGF-A protein, a non-translatable variant of AZD8601 (NT-VEGF-A mRNA), and citrate/saline vehicle. Furthermore, we dynamically evaluate the bioactivity of AZD8601 in an established mouse model of diabetic wound healing *in vivo*. Capitalizing on a boron nanoparticle-based tissue oxygen sensor^16^, we demonstrate the dose- and time-dependent effects of AZD8601 on the re-vascularization and re-oxygenation of the wound bed, which leads to accelerated re-epithelialization during the early phase of diabetic wound healing.

## Results

### Transfection efficiency, pharmacokinetic characterization, and hybridization of AZD8601

**Figure 1:**
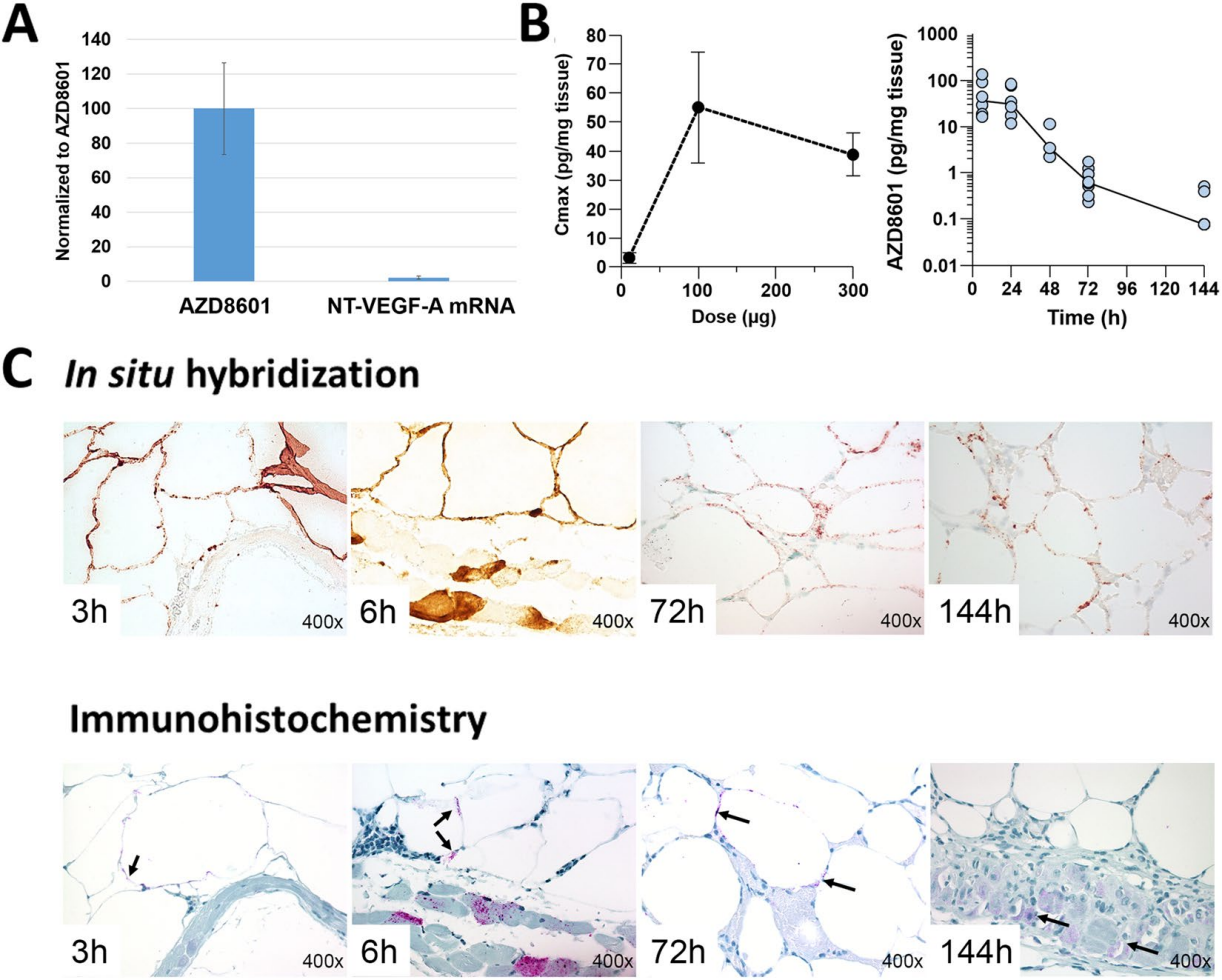
Transfection efficiency, pharmacokinetic characterization, and hybridization of AZD8601. **(A)** AZD8601 or Non-Translatable(NT)-VEGF-A mRNA was transfected into human aortic smooth muscle cells with Lipofectamine 2000 as transfection reagent. After 4 hours the transfection medium was removed and changed to fresh serum free media that was changed every 8 hours. At 24 hours the medium was collected and kept at −80 °C for subsequent measurement of human VEGF-A protein. Levels of VEGF-A protein produced are normalized to levels produced by AZD8601 (n = 3/group). **(B)** Maximum concentration of VEGF-A protein (Cmax) after intradermal injection in db/db mice of 10, 100 and 300 µg of AZD8601 in citrate/saline (n = 6/dose group, left panel). Human VEGF-A protein content in skin biopsies up to 144 hours after intradermal injection of 100 µg citrate/saline-formulated AZD8601 in db/db mice. The line represents the median at each time point (n = 3–8/time point, right panel). **(C)** *In situ* hybridization staining for AZD8601 (brown color in the upper panel) and immunohistochemistry staining for human VEGF-A protein (magenta color and black arrows in the lower panel) after intradermal injection of 100 µg AZD8601 in citrate/saline formulation as a function of time up to 144 hours. Magnification is 400x.

As shown in Fig. 1A, AZD8601 could be effectively transfected into human smooth muscle cells and led to the production of VEGF-A protein that was secreted into the supernatant. In contrast, transfection with NT-VEGF-A mRNA did not give rise to VEGF-A production.

Intradermal injection of AZD8601 formulated in citrate/saline to db/db mice (n = 6/dose group) showed that increased dosing of AZD8601 in the range of 10–100 µg led to increased maximum concentration (Cmax) of VEGF-A protein (Fig. 1B, left). However, increasing the dose of AZD8601 to 300 µg did not result in further increase in Cmax, suggesting saturation of VEGF-A production. Besides the dose dependence in protein production, we also evaluated the pharmacokinetics in the same *in vivo* setting. Our result showed that the concentration of VEGF-A protein in the db/db mouse skin (3–8 animals/time point assessed) reached a maximum of 36 pg/mg tissue at 6 hours after intradermal injection of AZD8601 (Fig. 1B, right). The total exposure (i.e., the area under the curve) of human VEGF-A protein over the entire 144-hour period was 1065 pg/mg tissue.

*In situ* hybridization and immunohistochemistry analysis of skin biopsies from db/db mice receiving 100 µg AZD8601 suggested that the mRNA and the produced VEGF-A protein could be found up to 144 hours post injection (Fig. 1C). The *in situ* hybridization analysis also showed that the cell types transfected in the mouse skin were mainly adipocytes and endothelial cells, with inflammatory, muscle and nerve cells transfected to a lesser extent (Fig. 1C). According to the immunohistochemistry analysis results, the most pronounced VEGF-A protein production was found in adipocytes followed by endothelial cells.

### Multifaceted microvascular response to AZD8601 *in vivo*

**Figure 2:**
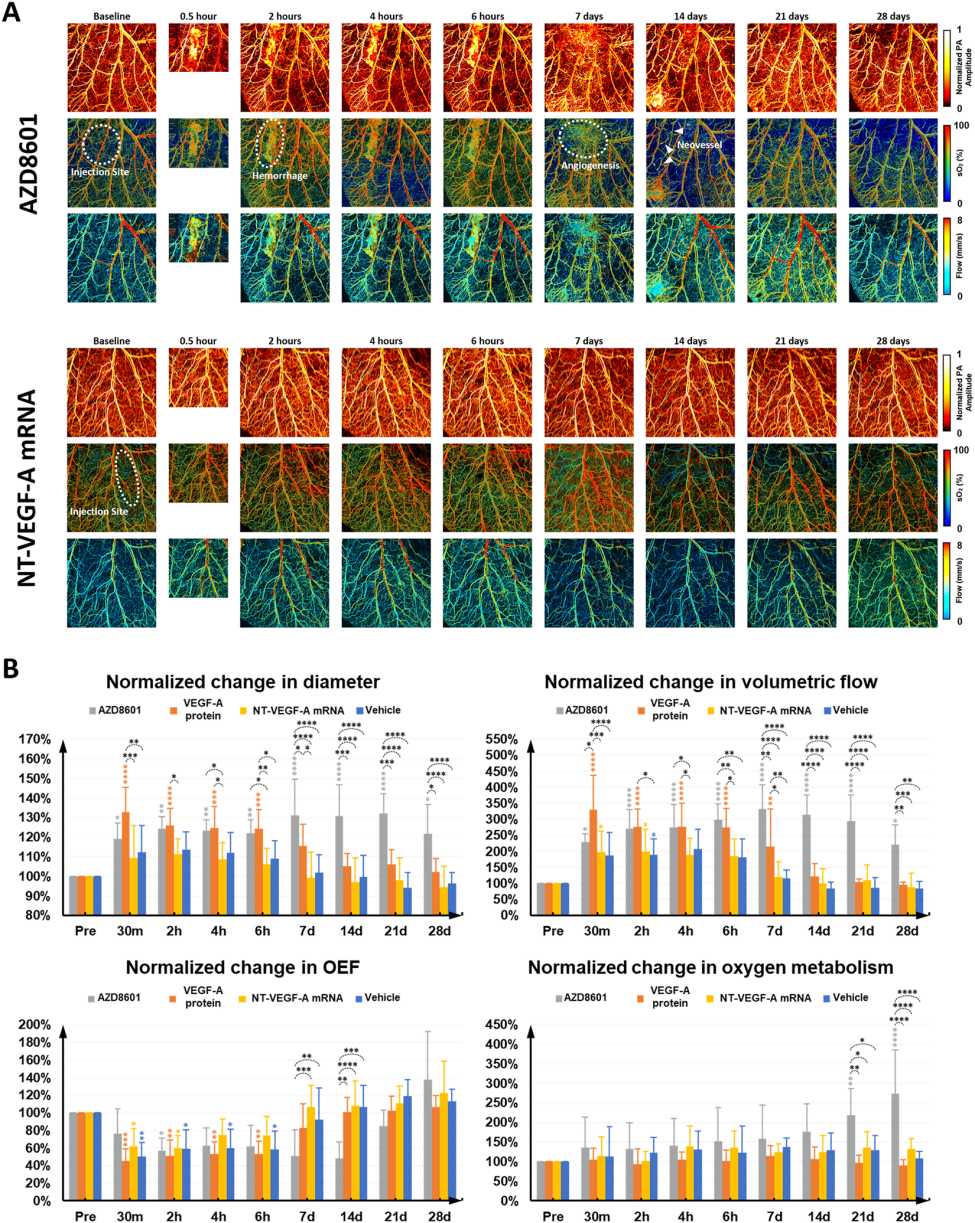
(**A**) Time-lapse multi-parametric photoacoustic microscopy of microvascular responses to 100 µg of AZD8601 or Non-Translatable(NT)-VEGF-A mRNA intradermally injected to the mouse ear. The top, middle and bottom rows respectively show the microvascular structure, sO_2_ and blood flow speed. PA: photoacoustic. **(B)** Statistical comparison of the multifaceted microvascular responses in diameter, volumetric flow, oxygen extraction fraction (OEF), and oxygen metabolism to AZD8601, VEGF-A protein, NT-VEGF-A mRNA, and citrate/saline vehicle (n = 8/group). *p < 0.05, **p < 0.01, ***p < 0.001, and ****p < 0.0001.

Following the biochemical characterization of AZD8601, we applied serial multi-parametric PAM to study the acute and chronic microvascular responses to AZD8601 in the mouse ear *in vivo*. Figure 2A and Supplementary Fig. 1 show representative time-lapse images of the microvascular structure, blood oxygenation (sO_2_), and blood flow in the ear intradermally injected with AZD8601, VEGF-A protein, NT-VEGF-A mRNA or citrate/saline vehicle. It is worth noting that all these microvascular measurements were performed in a label-free and noninvasive manner by exploiting the optical absorption of blood hemoglobin^17^.

With the aid of vessel segmentation^18^, we extracted these measurements on a single-vessel basis, from which we were able to quantitatively compare the microvascular responses to the four different compounds. As shown in Fig. 2B, significant increases in the vessel diameter and blood flow were observed shortly (30 minutes to 6 hours) after intradermal injection of AZD8601 or VEGF-A protein. In contrast, much weaker vasodilation and flow increase were observed in mice injected with NT-VEGF-A mRNA or citrate/saline, which was likely due to the needle invasion rather than the compound effect *per se*. The acute hemodynamic responses gradually regressed back to the baseline levels in the protein-injected group, but remained elevated throughout the entire 28-day monitoring period in the AZD8601 group. Interestingly, the dynamic changes in blood flow were tightly coupled with those in the oxygen extraction fraction (defined as $OEF=\frac{{s}_{a}{O}_{2}-{s}_{v}{O}_{2}}{{s}_{a}{O}_{2}}$, where *s*_*a*_*O*_2_ and *s*_*v*_*O*_2_ are arterial and venous *sO*_2_, respectively) in the three groups injected with VEGF-A protein, NT-VEGF-A mRNA or citrate/saline, resulting in a roughly constant metabolic rate of oxygen (estimated as *Blood flow* × *OFF*). In contrast, the oxygen metabolism of the AZD8601 group gradually increased over the 28-day time course, suggesting possible tissue proliferation due to the improved vascularization.

Besides the pronounced hemodynamic responses, striking angiogenesis and neovessel formation were also observed in some of the mouse ears injected with 100 µg AZD8601. As shown in the blowups of the blue boxed region (Supplementary Fig. 2, bottom row), densely packed new capillaries and two branches of neovessels (indicated by green arrows) appeared 7 and 14 days after the injection of AZD8601, respectively. The high brightness of these neovessels in the PAM images implies that they were highly perfused with red blood cells. Interestingly, the neovessels ‘regressed’ on day 21 and disappeared on day 28. The ‘regression’ and disappearance of these vessels was likely due to the loss of blood perfusion (the PAM signal comes from blood hemoglobin). In contrast, only moderate capillary angiogenesis but no neovascularization was observed in the VEGF-A protein group (Supplementary Fig. 1). Also, no angiogenesis or neovascularization was observed in the NT-VEGF-A mRNA and citrate/saline groups (Supplementary Fig. 1).

### Effects of sequential dosing of AZD8601 on the microvascular response

**Figure 3:**
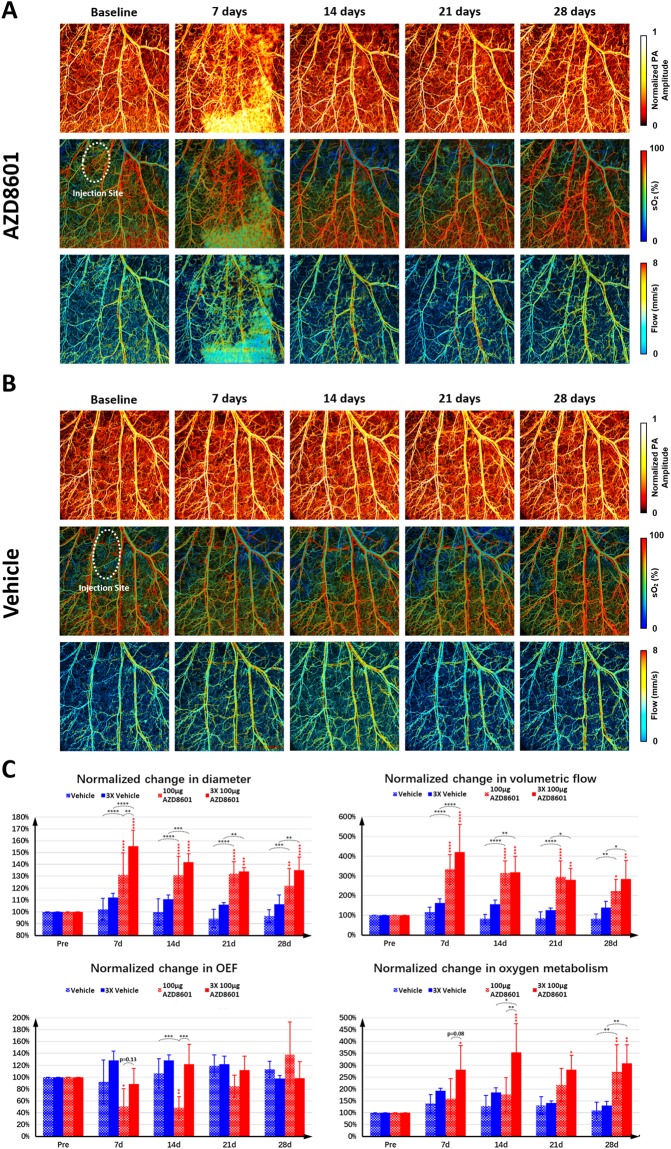
Time-lapse multi-parametric photoacoustic microscopy of microvascular responses to sequential dosing of (**A)** 100 µg AZD8601 or **(B)** saline vehicle intradermally injected to the mouse ear on day 0, 2 and 4. The top, middle and bottom rows respectively show the microvascular structure, sO_2_ and blood flow speed. PA: photoacoustic. **(C)** Statistical comparison of the multifaceted microvascular responses in diameter, volumetric flow, oxygen extraction fraction (OEF), and oxygen metabolism to single and multiple injections of AZD8601 and citrate/saline vehicle (n = 4/group). *p < 0.05, **p < 0.01, ***p < 0.001, and ****p < 0.0001.

To examine whether and how sequential dosing of AZD8601 could affect the microvascular response compared with single dosing, we repeated the time-lapse multi-parametric PAM monitoring experiment in mouse ears sequentially injected with 100 µg AZD8601 at the same location on day 0, 2 and 4 (Fig. 3A). Given the similar microvascular responses to NT-VEGF-A mRNA and citrate/saline (Fig. 2B), citrate/saline was used as the control in this study (Fig. 3B). As shown in Fig. 3C, sequential dosing of AZD8601 resulted in larger increases in the vessel diameter (p = 0.0034 on day 7), OEF (p = 0.0003 on day 14), and oxygen metabolism (p = 0.0038 on day 14), compared with the single-dosing group. The differential responses, however, leveled out after 21 days.

### Dose-dependent effects of AZD8601 on the microvascular response

In light of the dose-dependent protein production (Fig. 1B, left), we also investigated the dose dependence of the multifaceted microvascular response on AZD8601. Given the saturation effect beyond 100 µg, two lower doses of AZD8601 (30 and 10 µg) were studied. In mice injected with 30 µg AZD8601, a less sustained increase in sO_2_ and blood flow was observed, which regressed back to the baseline on day 14. Although capable of producing capillary angiogenesis around the injection site, neovessel formation was not observed (Supplementary Fig. 3). Reducing the AZD8601 dosage to 10 µg led to further compromised microvascular responses. Specifically, the increase in sO_2_ and blood flow was less significant and enduring (regressed to the baseline on day 7) and no angiogenesis or neovascularization was observed (Supplementary Fig. 4).

### Effects of sequential dosing of AZD8601 on the wound healing rate

**Figure 4:**
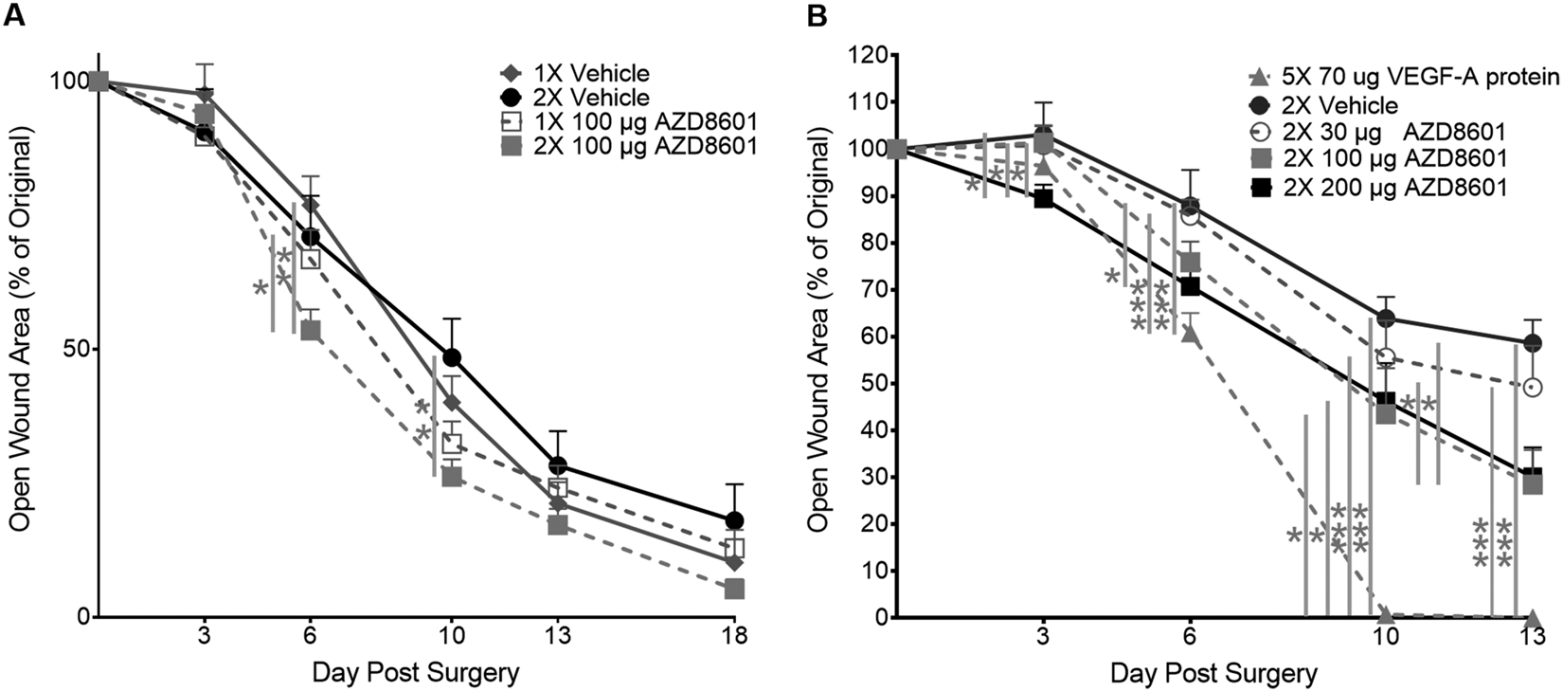
Repeated and higher doses of AZD8601 had greater effects on diabetic wound healing. **(A)** Dosing on days 0 and 3 increased early healing compared to dosing on day 0 only (n = 7–8/group). **(B)** The greatest effect on wound closure was achieved by the 200 µg dose of AZD8601, which came closest to replicating the effect of exogenous recombinant VEGF-A_165_ protein positive control (n = 6/group). *p < 0.05, **p < 0.01, ***p < 0.001.

Following the comprehensive characterization of the microvascular response to AZD8601, we studied the bioactivity and sequential dosing effect of AZD8601 in a mouse model of diabetic wound healing. As shown in Fig. 4A, a second dose of AZD8601 delivered on day 3 post-surgery (i.e., wounding) significantly increased early healing versus a single dose on day 0. Increased wound closure was observed in the double-injected AZD8601 group on days 6 and 10 post-surgery. On day 6, the double-injected AZD8601 group exhibited significantly smaller sizes of open wound (54 ± 3.9% of original area), compared with single-injected vehicle control (77 ± 5.4%) and double-injected vehicle control (71 ± 7.6%). On day 10, the double-injected AZD8601 group exhibited significantly smaller open-wound sizes (26 ± 3.3%) versus double-injected vehicle control (48 ± 7.3%).

### Dose-dependent effects of AZD8601 on the wound healing rate

Consistent with the dose-dependent microvascular response to AZD8601, higher doses of AZD8601 accelerated early wound healing (Fig. 4B). On day 3 post-surgery, the group treated with 200 µg AZD8601 exhibited significantly smaller open-wound sizes (89 ± 3.0%) versus the vehicle control (103 ± 6.9%). On day 6, both the 200 µg AZD8601 group (71 ± 3.6%) and the VEGF-A group (61 ± 4.2%) exhibited significantly smaller open-wound sizes versus the vehicle control (88 ± 7.7%). Likewise, on day 10, the 100 µg AZD8601 group (43 ± 9.8%), the 200 µg AZD8601 group (46 ± 8.2%), and the VEGF-A group (0.7 ± 0.3%) exhibited significantly smaller open wounds versus the vehicle control (64 ± 4.6%). On day 13, the 100 µg AZD8601 group (28 ± 7.4%), the 200 µg AZD8601 group (30 ± 6.3%), and the VEGF-A group (0 ± 0.0%) all exhibited significantly increased wound closure versus the vehicle control (59 ± 5.0%).

### Wound oxygenation monitoring with boron nanoparticle-based tissue oxygen sensor

**Figure 5:**
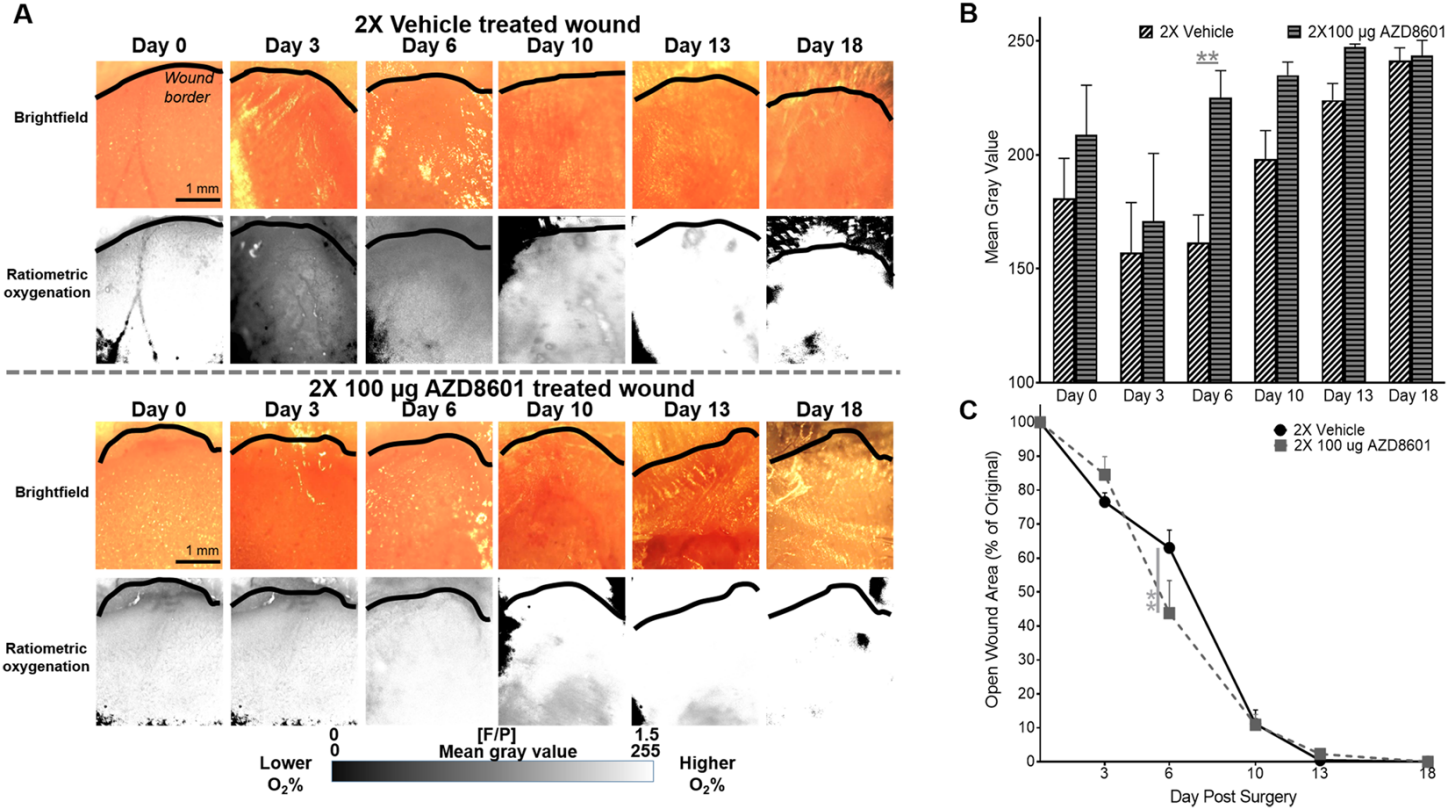
AZD8601 treatment resulted in increased oxygenation of diabetic wound beds during healing. **(A)** Black and white ratiometric images of fluorescence/phosphorescence signal from BF_2_nbm(I)PLA (Difluoroboron β-diketonate poly(lactic acid)) nanoparticles revealed increased oxygenation in AZD8601-treated wounds. **(B)** Wound oxygenation as calculated by mean gray value of ratiometric images was significantly increased at day 6 post-surgery in the AZD8601-treated group. **(C)** Addition of BF_2_nbm(I)PLA nanoparticles to wounds did not impair healing. **p < 0.01, n = 3–4/group.

Besides the wound healing rate, we also evaluated the tissue oxygenation of the wound bed using the boron nanoparticle-based tissue oxygen sensor^16^ (Fig. 5A). As shown in Fig. 5B, the tissue oxygenation level of the wound bed, estimated by the mean gray value of the black and white fluorescence/phosphorescence ratiometric outputs, was significantly increased on day 6 post-surgery in the double-injected AZD8601 group (225 ± 11.9%) as compared to the double-injected vehicle control (161 ± 12.2%). The improved tissue oxygenation was accompanied by the smaller open-wound size in the double-injected AZD8601 group (44 ± 9.6%) versus the double-injected vehicle control group (63 ± 5.2%) on day 6 (Fig. 5C).

## Discussion

Capable of mediating efficient and localized expression of VEGF-A without eliciting innate immune responses, AZD8601 is ideally suited for VEGF-A therapies in a spatiotemporally confined manner and with low toxicity^10,11^. By promoting vascular remodeling and neovessel formation, this emerging therapeutic holds great potential in treating diabetic wound and cardiovascular diseases^19^. However, the *in vivo* efficacy and dosing pharmacokinetics of AZD8601 still remain incompletely understood.

Here, we dynamically, quantitatively and comprehensively examined the effects of AZD8601 on the vascular structure, function and associated oxygen supply/metabolism at the microscopic level *in vivo*, using multi-parametric PAM. Side-by-side comparison of the multifaceted microvascular response to AZD8601, VEGF-A protein, NT-VEGF-A mRNA, and citrate/saline vehicle showed pronounced, sustained, dose-dependent and AZD8601-specific vasodilation, blood flow increase, oxygen-metabolic upregulation, angiogenesis and neovessel formation (Fig. 2). Sequential dosing of AZD8601 at the same tissue location on day 0, 2 and 4 further boosted the early responses (i.e., day 7 or 14) in vasodilation and oxygen extraction/metabolism compared with single dosing, which, however, leveled out after 21 days (Fig. 3). Reducing the AZD8601 dose from 100 µg to 30 or 10 µg resulted in much compromised and less enduring microvascular remodeling, along with abolished neovascularization (Supplementary Fig. 3 and Supplementary Fig. 4).

Promoting sustained vascularization and blood oxygen supply *in vivo*, AZD8601 demonstrated beneficial therapeutic effects on diabetic wound healing. In the established mouse model of diabetic wound, we showed that intradermal injection of AZD8601 after wounding significantly accelerated re-epithelization relative to administering citrate/saline vehicle. Sequential dosing of AZD8601 on days 0 and 3 increased early healing compared to single dosing on day 0. Further, the efficacy of AZD8601 was benchmarked to that of VEGF-A protein (Fig. 4), which has previously been shown to accelerate vascularization and wound healing in diabetic wounds^20^. In addition to the standard open-wound analysis, time-lapse monitoring of the tissue oxygenation within the wound area using the boron nanoparticle-based tissue oxygen sensor^16^ revealed a strong correlation between the oxygenation level and the open-wound size, suggesting that the accelerated wound healing can be attributed, at least partially, to the AZD8601-facilitated re-oxygenation of the wound (Fig. 5).

It is worth pointing out that there is a high degree of coherence between the observations on the AZD8601-induced microvascular responses in the intact ear and the AZD8601-facilitated healing process in the diabetic wound. First, the significantly improved re-oxygenation of the wound in the AZD8601-treated mice nicely echoes the neovascularization and oxygen-metabolic upregulation induced by AZD8601 in the mouse ear. Second, increased dosage of AZD8601 resulted in both more pronounced microvascular responses in the ear (Supplementary Figs 2–4) and more rapid healing of the wound (Fig. 4B). Last but not the least, sequential dosing of AZD8601 led to more significant early microvascular responses versus single dosing in the ear, which, however, leveled out after 21 days (Fig. 3); this observation again nicely echoes that on the wound healing process, where sequential dosing of AZD8601 sped up the healing but did not lead to better end results compared with single dosing (Fig. 4A). Together, these observations support a bioactive effect for AZD8601, whereby the microvascular remodeling and oxygen-metabolic upregulation contributes to accelerated/improved tissue regeneration.

Relative to other pharmacological and cell-based treatments that have been shown to favorably impact re-epithelialization in murine models of delayed diabetic wound healing, AZD8601 induced comparable levels of wound healing acceleration^21^. However, treatment with AZD8601 is less expensive than stem cell therapy, and immunological rejection is not a complicating factor. Moreover, unlike treatment with VEGF-A protein, AZD8601 did not induce edema or the formation of micro-hemangiomas, as evidenced by a red blushing of the wound in our study (Supplementary Fig. 5). Since sequential topical dosing of recombinant human VEGF-A_165_ protein to chronic diabetic neuropathic foot ulcers has been clinically shown to reduce the time to complete ulcer healing^22^, we expect that the clinical evaluation of AZD8601 in diabetic wounds could reveal a superior benefit to diabetic wound healing by promoting functional angiogenesis in the absence of edema and micro-hemangioma formation^23^.

We caution against comparing the effects of recombinant VEGF-A protein on healing rates in the diabetic wound model (Fig. 4B) to its effects on volumetric blood flow, diameter, and tissue oxygenation in the healthy ear model (Fig. 2) because the dose of VEGF-A protein delivered to the diabetic wound was 72-fold higher than that delivered to the ear. Furthermore, the ear received injections of VEGF-A protein at three time points (day 0, 2 and 4; Fig. 3), while the wound received topical applications of VEGF-A protein at five time points (day 0, 2, 4, 6 and 8 post wounding; Fig. 4). Because the VEGF-A protein-treated group served as the positive control in the wound healing study, the dose and dosing time course of VEGF-A protein were based on previously published data^20,22^. Based on the observed changes in the diabetic wounds treated with VEGF-A protein, we believe that this dose of VEGF-A protein is essentially an over-treatment that would not have been useful or plausible to repeat in the ear of healthy mice.

In summary, we demonstrate the pronounced, sustained and dose-dependent effects of AZD8601 on the microvasculature *in vivo* and its benefits to diabetic wound healing, including improved tissue oxygenation and accelerated healing rate without edema and micro-hemangioma. Out data provide strong preclinical evidence to support the potential clinical translation of this promising therapeutic^23^. Also, it is worth noting that it is the two innovative technologies that enable us to comprehensively evaluate the effects of AZD8601 on both blood oxygenation (PAM) and tissue oxygenation (boron nanoparticle-based oxygen sensor) in a noninvasive and longitudinal manner *in vivo*. Future integration of the two enabling technologies into a dual-modal system will open up new opportunities in cardiovascular research.

## Methods

### mRNA synthesis and formulation

AZD8601 was synthesized as described elsewhere^13^.

### Study design

The central goal of this study was to comprehensively characterize the microvascular response to AZD8601 and evaluate its bioactivity in diabetic wound healing *in vivo*. To this end, we employed two state-of-the-art technologies: multi-parametric PAM and boron nanoparticle-based tissue oxygen sensing. First, we quantified the acute (30 minutes to 6 hours post-injection) and chronic (up to 28 days) responses of the microvascular structure and function, as well as the associated oxygen extraction and metabolism, to AZD8601 intradermally injected in the mouse ear. Then, we compared the multifaceted response to AZD8601 with those to VEGF-A protein, NT-VEGF-A mRNA, and citrate/saline vehicle (n = 8/group). In parallel, we studied the dose dependence of the microvascular response on AZD8601. The PAM-based *in vivo* characterization was compared with the transfection efficiency, pharmacokinetics and hybridization of AZD8601 characterized by biochemical assays. Following the characterization, we evaluated the bioactivity of AZD8601 in a mouse model of diabetic wound healing in three separate trials. Trial 1 (n = 7–8/group) was designed to address the influence of single (i.e., dosing on day 0 only) or double (dosing on day 0 and day 3, respectively) dosing. Trial 2 (n = 3–4/group) combined the boron nanoparticle-based tissue oxygen sensor and standard wound healing analysis protocol to examine the effects of AZD8601 on the re-oxygenation of the wound bed and the healing rate. Trial 3 (n = 6/group) was designed as a dose-response study (30, 100 and 200 µg AZD8601), including a positive control (recombinant VEGF-A_165_ protein).

### Transfection efficiency in human aortic smooth muscle cells *in vitro*

The transfection potential of both AZD8601 and NT-VEGF-A mRNA (Moderna Therapeutics) were assessed in human aortic smooth muscle cells. Ten thousand cells were seeded into 96-well plates in smooth muscle growth medium (SmGM-2; Lonza). In the following day, transfection was undertaken in the serum-free medium. Following the manufacturer’s instruction, 250 ng of AZD8601 or NT-VEGF-A mRNA (Moderna Therapeutics) was mixed with Lipofectamine 2000 and then added to the cells. After 4 hours, the transfection medium was removed and changed to fresh serum-free media that was collected after 24 hours and kept at −80 °C. The experiment was repeated three times. The amount of VEGF-A protein in the supernatant post transfection was measured using a human VEGF-A ELISA kit (Novex, Invitrogen). The absorbance was measured at 450 nm using a SpectraMax reader (Molecular Devices).

### *In situ* hybridization for AZD8601 and immunohistochemistry for VEGF-A protein in skin

At predefined time points after intradermal injection of AZD8601 on the back of male db/db mice, skin biopsies with an area of 0.785 mm^2^ was taken from the back of male db/db mice using a 10-mm biopsy punch. The biopsy was put in 4% formaldehyde for immersion fixation and cut in three slices. Subsequently, the pieces were embedded in paraffin and further sectioned into 4-µm slices. RNAscope-automated *in situ* hybridization assay for detection of AZD8601 was carried out in the Bond RX platform (Leica Biosystems) and all *in situ* hybridization reagents were products of the Advanced Cell Diagnostics. Briefly, target retrieval was performed at 95 °C for 15 minutes using Leica Epitope Retrieval Buffer 2 followed by protease treatment at 42 °C for 15 minutes. The probe (RNAscope LS2.5 Probe-Hs-VEGFA-noXrodent cat. nr 412018; Advanced Cell Diagnostics) were hybridized for 2 hours at 42 °C followed by RNAscope amplification, and 3,3′-diaminobenzidine was used to visualize staining. Immunohistochemistry for detection of VEGF-A protein was carried out in the Ventana discovery Ultra immunostainer, and all reagents were Ventana products (Roche). Antigen retrieval was done in Ventana Cell Conditioner 1 for 24 minutes at 95 °C. VEGF-A primary antibody was added for 1 hour at 37 °C (dilution 1:100, Cat. # RB-9031-P0; Thermo Fisher Scientific), followed by secondary anti-rabbit HQ reagent and anti-HQ HRP Purple chromogenic detection.

### Quantification of VEGF-A protein in mouse skin

At predefined time points after intradermal injection of AZD8601 on the back of male db/db mice, tissue biopsies from the injection site on the back or the entire ear was sampled and snap frozen in liquid nitrogen and stored at −80 °C until processed. Tris lysis buffer containing phosphatase inhibitors I and II and protease inhibitor (R60TX; Meso Scale Diagnostics) was added to the frozen tissue biopsies and kept at −20 °C prior to homogenization. Stainless steel beads (2.8 mm) were then added, and the samples were homogenized using the Precellys homogenizer. The homogenates were centrifuged, and the supernatants were stored at −80 °C pending analysis. Concentrations of VEGF-A_165_ were determined using a sandwich immunoassay with electrochemical luminescent detection. V-PLEX Human VEGF assay kit (K151RHD, Meso Scale Diagnostics) was used to measure the VEGF-A_165_ concentration in the tissue homogenates. Standards were serially diluted in MSD diluents. Samples with high concentration were diluted with MSD diluents prior to analysis to fit within the standard curve, and the plates were read on the MSD’s Sector Imager 6000.

### Multi-parametric PAM of the mouse ear microvasculature

Six-week-old C57BL/6BrdCrHsd-*Tyr*^*c*^ mice (Envigo) were used for this study. Multi-parametric PAM of the microvascular response in the mouse ear is completely label-free and noninvasive. Thus, the same ear was repeatedly imaged for time-lapse monitoring of drug effects (100 µg AZD8601, 100 µg NT-VEGF-A mRNA, 1 µg recombinant VEGF-A_165_ protein (R&D Systems), and 10 µL citrate/saline vehicle) on the microvascular diameter, sO_2_, blood flow, angiogenesis and neovascularization over a prolonged period of 28 days.

Prior to intradermal injection of the drugs (Supplementary Movie 1), a set of baseline images of the mouse ear were acquired using multi-parametric PAM. Then, the drug-treated ear was repeatedly imaged for 6 hours to capture acute microvascular responses and reimaged on a weekly basis to record chronic responses and possible angiogenesis and/or neovascularization. In cases of multiple injections, the drugs were injected at the same location in the ear on day 0, 2 and 4.

To avoid motion artifacts, PAM of the mouse ear was conducted under general anesthesia, which was inducted with 2% isoflurane vaporized by medical-grade air (Praxair) at a flow rate of 1–1.5 L/min. After the induction of anesthesia, the mouse was transferred to a nearby stereotaxic stage in the PAM system, and a thin layer of ultrasonic gel (Parker) was gently applied to the ear surface for ultrasound coupling. Care was taken to avoid trapping air bubbles inside the gel. Then, the ear was placed beneath a container filled with temperature-controlled deionized water (37 °C) and slowly raised until the gel was in gentle contact with the bottom of the container, which was covered by a thin membrane of polyethylene (S. C. Johnson & Son). The imaging head of the PAM was then lowered and immersed in the water container. Air bubbles trapped under the imaging head were removed. Throughout the PAM experiment, the mouse was maintained under general anesthesia with 1.5% isoflurane and its body temperature was kept at 37 °C using a heating pad (SRFG-303/10; Omega) and a temperature controller (EW-89802-52; Cole-Parmer). Ointment was applied to the mouse eyes to prevent drying and accidental laser damage. The laser fluence was carefully controlled to comply with the safety standards of the American National Standards Institute (i.e., 20 mJ/cm^2^). At the conclusion of the experiment, the mouse ear was cleaned with deionized water before being transported back to its home cage.

Three microvascular parameters (i.e., structure, sO_2_ and blood flow) were simultaneously acquired by multi-parametric PAM, which was reported before^15^. Briefly, the vascular structure was directly generated by Hilbert transforming the raw PAM signals. The vascular sO_2_ was acquired with dual-wavelength (532 and 559 nm) laser excitation to distinguish oxy- and deoxy-hemoglobin via their optical absorption spectra. The blood flow speed was quantified by correlating 100 successive PAM A-lines acquired at 532 nm. Further, the average diameter, sO_2_ and blood flow of individual vessels were extracted using our self-developed vessel segmentation algorithm^18^, with which volumetric blood flow, OEF and oxygen metabolism can be derived. Eight mice were studied per group, and the measurements of different animals within each group were combined for statistical analysis.

### Assessment of diabetic wound healing

Eight-week-old B6.BKS(D)-*Lepr*^*db*^/J mice (Jackson Laboratory) were used for the wound healing study. Prior to wounding, mice were fasted for 4 hours on wood chip bedding with access to water. Initial fasted glucose measurements were taken on blood drawn from tail veins. At the terminal endpoint of the study and prior to tissue harvest, mice were anesthetized via inhalation of 2% isoflurane/98% oxygen mixture and fed glucose measurements were taken from blood obtained via cardiac puncture.

Mice were anesthetized by inhalation of 2% isoflurane/98% oxygen mixture. Dorsa were shaved and depilated before being sterilized with three alternating scrubs of povidone-iodine and 70% isopropanol. Full-thickness cutaneous wounds, which were 1 cm in diameter, were surgically made on the dorsum of each mouse. An analgesic (buprenorphine, 0.1 mg/kg) was administered following the surgery and the wounds were covered with a Tegaderm dressing. Mice were singly housed with food and water available *ad libitum*.

Ten μL of AZD8601 (0.75, 2.5, or 5 mg/mL in 10 mmol/L citrate/130 mmol/L saline) or citrate/saline vehicle (10 mmol/L/130 mmol/L) were injected intradermally at four equidistant points around the wound edge. Study groups indicated as “single-injected” received injections on day 0 only. Study groups indicated as “double-injected” received injections on day 0 and 3. The injections were placed at the 0, 90, 180, and 270 degree positions on day 0. Injections administered on day 3 were placed at the 45, 135, 225, and 315 degree positions (Supplementary Fig. 6). Seventy-two μL of recombinant VEGF-A protein (1 μg/μL in 0.9% saline with 0.1% bovine serum albumin; R&D Systems) was administered topically to the wounds of one treatment group. This group received VEGF-A delivery at 0, 2, 4, 6, and 8 days post-surgery.

For Bright-field imaging, Tegaderm dressings were removed when mice were anesthetized. Wounds were illuminated with a Dolan-Jenner MI 150 fiber optic illuminator (Edmund Optics) and photographed with an iPhone 6 (Apple) mounted to a clamp and affixed to a ring stand. Wounds were imaged on days 0, 3, 6, 10, 13 (all trials), and 18 (trials 1 and 2 only).

For oxygen-sensing nanoparticle imaging, a Grasshopper3 camera (GS-U3-41C6C; FLIR) was mounted to a microscope (Eclipse 80i; Nikon) equipped with a 4X objective, a fluorescence light source (X-Cite 120Q; Excelitas Technologies), a bandpass excitation filter (360/20 nm), and a long pass barrier filter ( >425 nm). Images were acquired with Flycapture2 software (FLIR). Mice were anesthetized via inhalation of 2% isoflurane and Tegaderm bandages were removed. The body temperature of the mouse was maintained throughout imaging, and wounds were irrigated with sterile 0.9% saline solution when necessary to prevent drying. Fifty μL of BF_2_nbm(I)PLA (Difluoroboron β-diketonate poly(lactic acid))^16^ nanoparticle solution was added to the wound (when necessary, a piece of sterile gauze was used to wick saline solution from the wound immediately prior to addition of the nanoparticle solution), and fluorescent and phosphorescent signals were recorded. Nanoparticle imaging was performed prior to addition of treatments, and nanoparticle solution was flushed from the wound with sterile 0.9% saline following imaging.

The UV-illuminated wound images were analyzed using a self-developed MATLAB program. Background signals in the blue and red channels were calculated from wound images taken prior to the addition of nanoparticle solution. These background values were subtracted from red and blue intensity values acquired after the addition of the nanoparticles. The ratio of blue channel intensity to red channel intensity was computed for each pixel to represent the ratio of fluorescence (constant in the presence of BF_2_nbm(I)PLA) to phosphorescence (quenched in the presence of oxygen). To map the relative amount of oxygen within the wound bed, we used the ratio of the background-subtracted blue to red channel intensity values to construct a grayscale image (low oxygen: black; high oxygen: white). We selected the wound bed as the region of interest and quantified the mean gray pixel value using ImageJ (National Institutes of Health).

Mice were euthanized under general anesthesia by exsanguination via cardiac puncture followed by CO_2_ asphyxiation. Approximately 1-mL blood was collected from each mouse into a BD Microtainer MAP (Becton Dickinson) and spun at 1,000 × g for 10 minutes at 4 °C. Plasma was isolated after centrifugation and stored in low-protein-retention micro centrifuge tubes. A 1.5 × 1.5 cm^2^ area of skin around the wound center was excised and snap frozen in liquid nitrogen for analysis.

### Statistics

To quantitatively compare the differences between the multifaceted microvascular responses to AZD8601, NT-VEGF-A mRNA, VEGF-A protein, and citrate/saline vehicle as shown in Figs 2B, 3C, 4 and 5B, a two-way ANOVA analysis was used. For multiple comparison, Tukey correction was used to control the Type I error rate differences. In all studies, mice were randomly assigned to AZD8601, NT-VEGF-A mRNA, VEGF-A protein, and citrate/saline groups. The number of mice in each group were chosen by power analysis and previous experience.

### Study approval

All experimental protocols were approved by Animal Care and Use Committee at the University of Virginia, US, and the local ethics committee on animal experiments in Gothenburg (district of Vastra Gotaland and Varmland). All animal procedures performed in this study were in accordance with Animal Care and Use Committee at the University of Virginia, US, and the local ethics committee on animal experiments in Gothenburg (district of Vastra Gotaland and Varmland) and in accordance with the animal welfare policy of Astra Zeneca.

## Electronic supplementary material

Supplementary Movie 1. Intradermal injection procedures

Supplementary Materials

## Data Availability

The datasets generated and analyzed during the current study are available from the corresponding author on reasonable request.
