# Supplementary material for: Modified VEGF-A mRNA induces sustained multifaceted microvascular response and accelerates diabetic wound healing
Source: Sci Rep. 2018 Nov 30;8:17509. doi: 10.1038/s41598-018-35570-6 (PMC6269526; doi:10.1038/s41598-018-35570-6)
Supplement: Supplementary file 2 — Supplementary Materials [file 41598_2018_35570_MOESM2_ESM.pdf]

Supplementary Information

Modified VEGF-A mRNA induces sustained multifaceted microvascular response and accelerates diabetic wound healing

Authors

Naidi Sun[1], Bo Ning[1], Kenny Hansson[2], Anthony C. Bruce[1], Scott A. Seaman[1], Chenchu Zhang[1], Michaela Rikard[1], Christopher A. DeRosa[3], Cassandra L. Fraser[3], Maria Wågberg[2], Regina Fritsche-Danielson[4], Johannes Wikström[2], Kenneth R. Chien[5,6], Anna Lundahl[7], Mikko Hölttä[8], Leif Carlsson[2], Shayn M. Peirce[1*], Song Hu[1*]

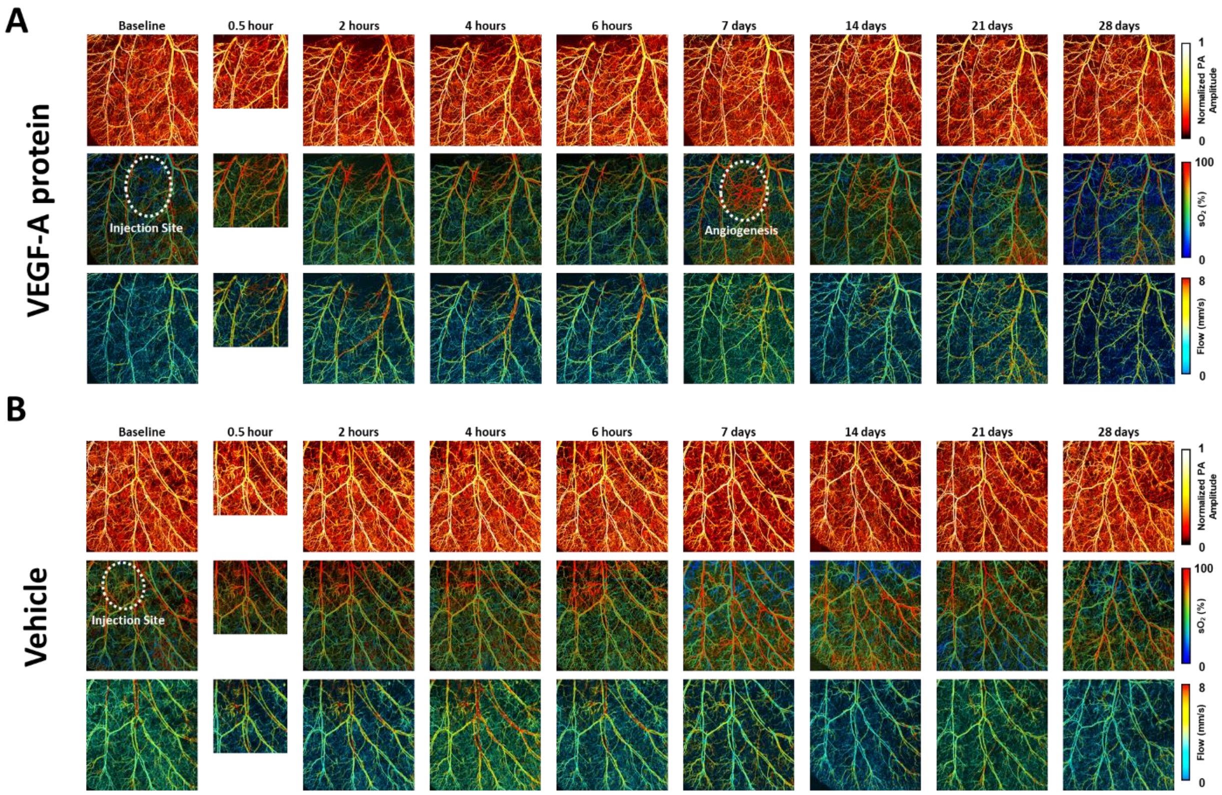

Supplementary Figure 1. Time-lapse multi-parametric photoacoustic microscopy of microvascular responses to **(A)** recombinant human VEGF-A protein or **(B)** citrate/saline vehicle intradermally injected to the mouse ear. The top, middle and bottom rows respectively show the microvascular structure, sO_2 and blood flow speed. PA: photoacoustic.

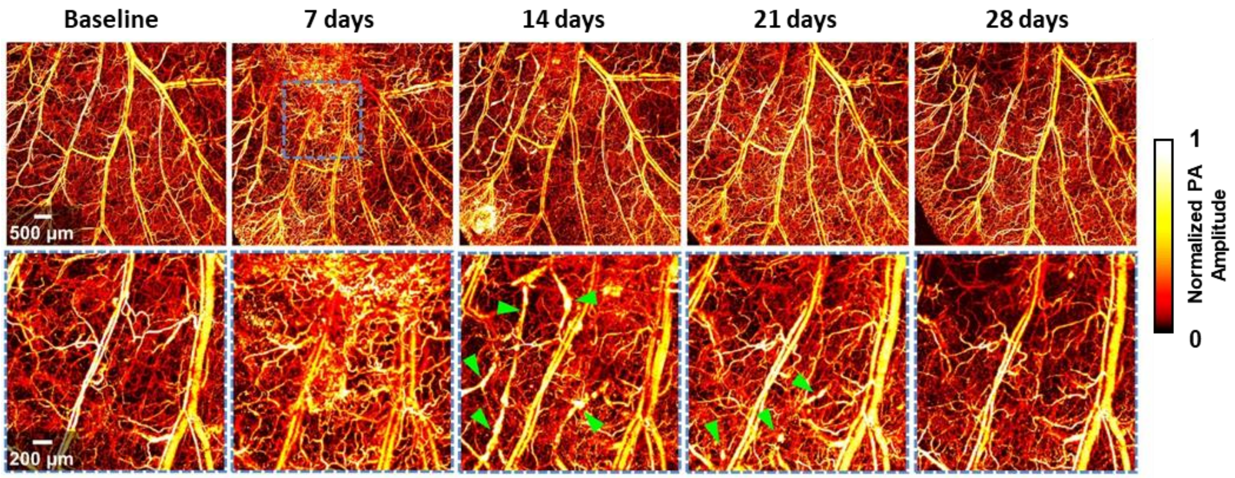

Supplementary Figure 2. Intradermal injection of 100 µg AZD8601 induces angiogenesis and neovascularization. Top row, microvascular structure of the whole field of view. Bottom row, zoom-in of the blue boxed region showing detailed view of the capillary angiogenesis on day 7 and neovessel formation on day 14 (green arrows). PA: photoacoustic.

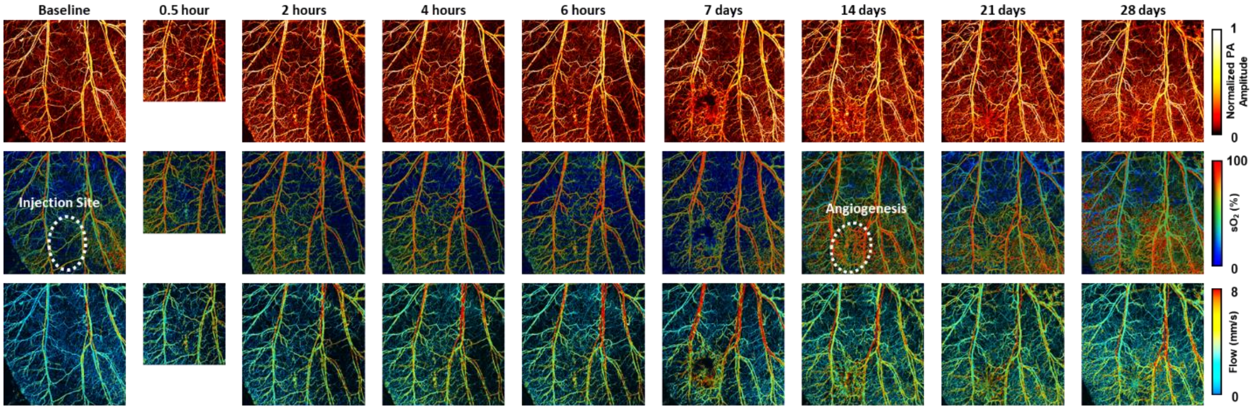

Supplementary Figure 3. Time-lapse multi-parametric photoacoustic microscopy of microvascular responses to 30 µg of AZD8601 intradermally injected to the mouse ear. The top, middle and bottom rows respectively show the microvascular structure, sO_2 and blood flow speed. PA: photoacoustic.

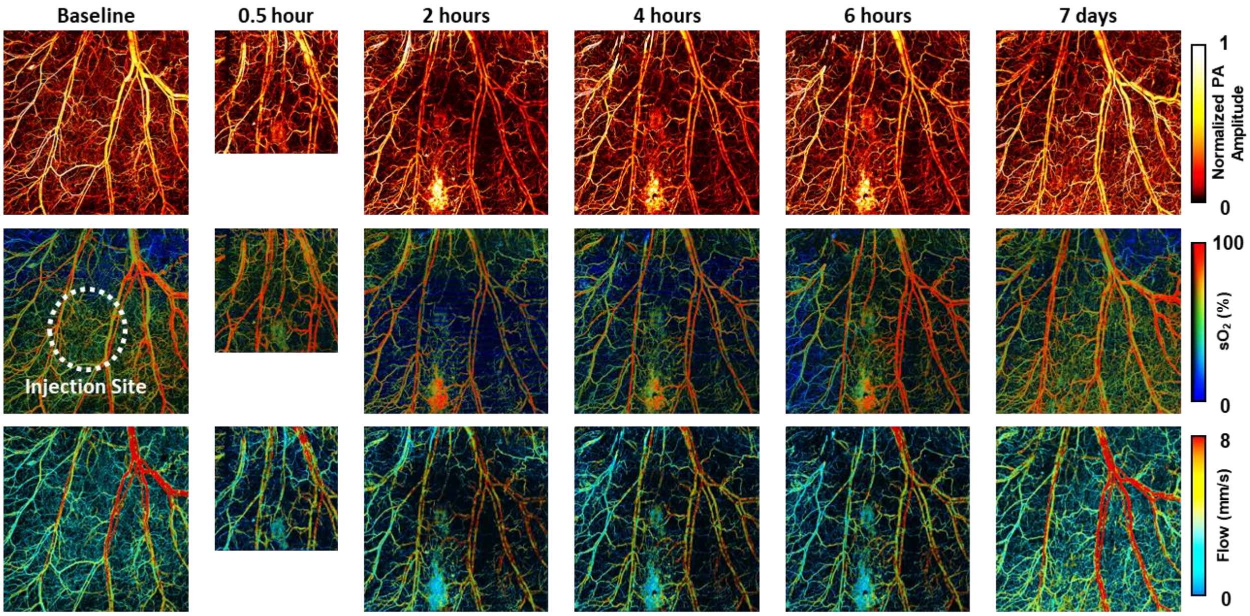

Supplementary Figure 4. Time-lapse multi-parametric photoacoustic microscopy of microvascular responses to 10 µg of AZD8601 intradermally injected to the mouse ear. The top, middle and bottom rows respectively show the microvascular structure, sO_2 and blood flow speed. PA: photoacoustic.

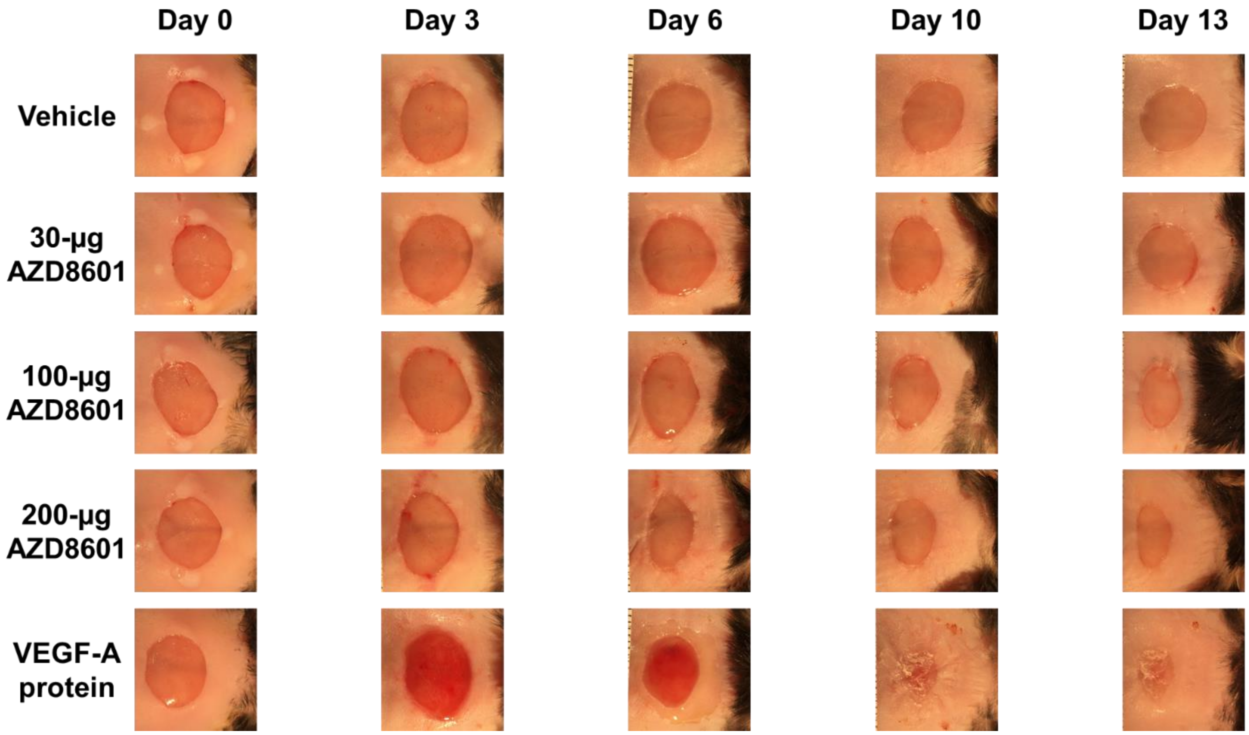

Supplementary Figure 5. Treatment using recombinant human VEGF-A_{165} protein induced a red coloration in healing wound beds. The 200 µg dose of AZD8601 (administered on Days 0 and 3) came closest to replicating the effect of exogenous VEGF-A_{165} protein positive control on wound closure. However, AZD8601 treatment did not result in the same consistent red coloration evident in VEGF-A_{165} protein-treated wounds (administered on Day 3 and 6).

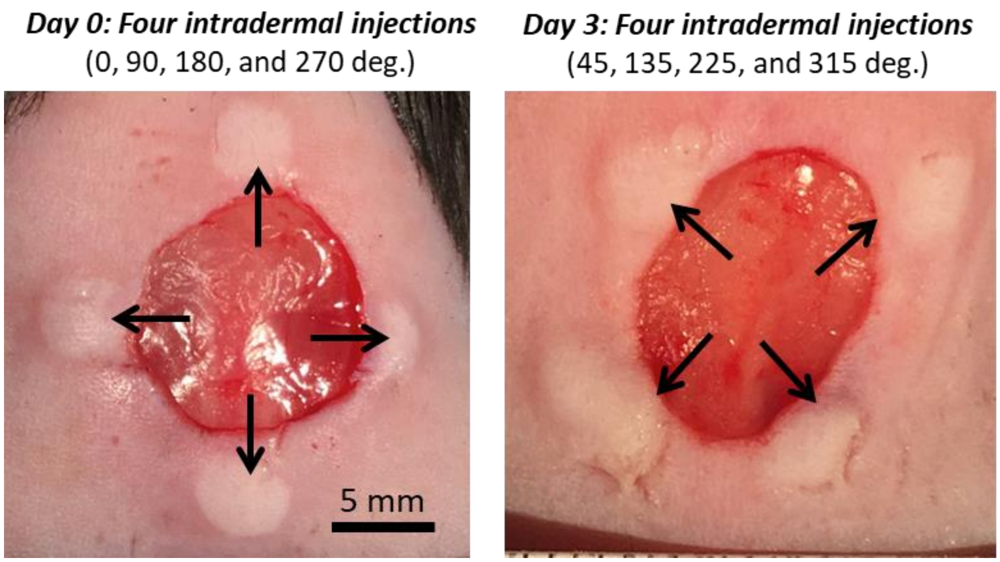

Supplementary Figure 6. Intradermal injection placement. AZD8601 and vehicle control were administered intradermally around the perimeter of the wound. Injections were placed at the 0, 90, 180, and 270 degree positions on day 0. Injections administered on day 3 were placed at the 45, 135, 225, and 315 degree positions.

Supplementary Movie 1. Intradermal injection procedure.

1) Prepare an ear holder: a metal cone (Wilton round tip 12, Walmart) with the back side horizontally inserted into a piece of modeling clay for stabilization.

2) The mouse is anesthetized and placed under a stereomicroscope.

3) Before injection, the cone is coated with a piece of double-side tape.

4) Then, the mouse ear is cleaned with ethanol, air-dried, and attached to the cone via the tape.

5) Use the stereomicroscope to guide the insertion of a 30-gauge syringe near the base of the ear pinna. Slowly advance the needle toward the center of the pinna.

6) Once the needle tip reaches the desired delivery site, inject the solution.

7) Hold the needle in position for 10–20 seconds before withdrawing it.
